# Preparation and Properties of Indium Ion Modified Graphite Felt Composite Electrode

**DOI:** 10.3389/fchem.2022.899287

**Published:** 2022-04-27

**Authors:** Yang Su, Na Chen, Hai-lin Ren, Li-li Guo, Zhen Li, Xiao-min Wang

**Affiliations:** ^1^ Liaoning Key Laboratory of Chemical Additive Synthesis and Separation, School of Materials Science and Engineering, Yingkou Institute of Technology, Yingkou, China; ^2^ School of Materials Science and Metallurgy, University of Science and Technology Liaoning, Anshan, China

**Keywords:** iron-chromium flow battery, graphite felt, indium ion, specific surface area, electrochemical performance

## Abstract

Iron-chromium redox flow batteries (ICRFBs) have the advantages of high safety, long cycle life, flexible design, and low maintenance costs. Polyacrylonitrile-based graphite felt composite material has good temperature resistance, corrosion resistance, large surface area and excellent electrical conductivity, and is often used as the electrode material of ICRFB, but its chemical activity is poor. In order to improve the activity of the graphite felt electrode, In^3+^ was used for modification in this paper, and the modified graphite felt was used as the electrode material for iron-chromium batteries. The structure and surface morphology of the modified graphite felt were analyzed by the specific surface area analyzer and scanning electron microscope; the electrochemical impedance spectroscopy and cyclic voltammetry experiments were carried out on the electrochemical workstation to study the electro catalytic activity of In^3+^ modified graphite felt and its performance in ICRFBS. The results show that the graphite felt electrode modified with a concentration of 0.2 M In^3+^ was activated at 400°C for 2 h, and its surface showed a lot of grooves, and the specific surface area reached 3.889 m^2^/g, while the specific surface area of the untreated graphite felt was only 0.995 m^2^/g significantly improved. Electrochemical tests show that the electrochemical properties of graphite felt electrodes are improved after In^3+^ modification. Therefore, the In^3+^ modified graphite felt electrode can improve the performance of ICRFB battery, and also make it possible to realize the engineering application of ICRFB battery.

## Introduction

**FIGURE 1:**
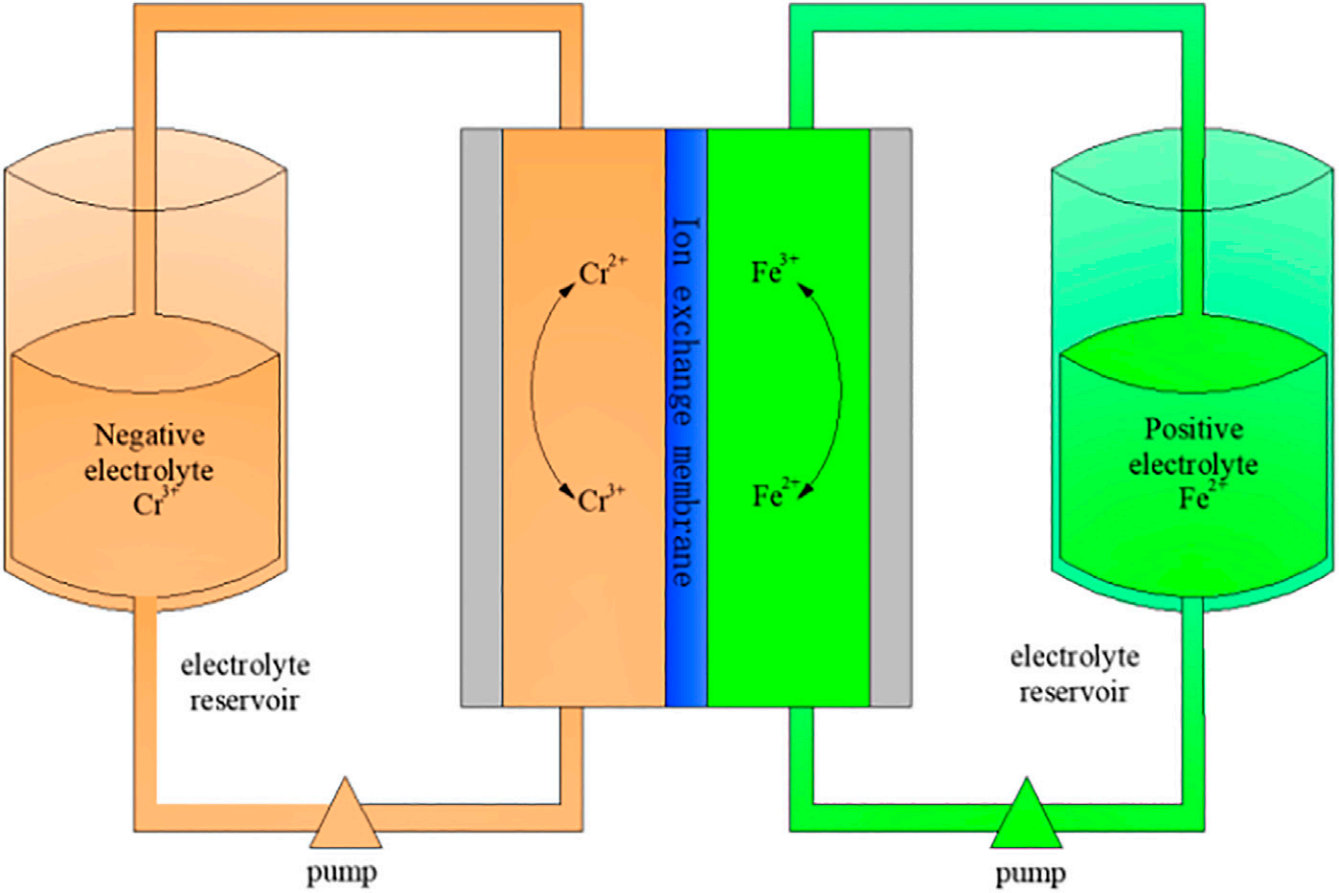
Composition diagram of iron-chromium flow battery.

In recent years, with the depletion of non-renewable resources such as coal, oil, and natural gas, renewable energy such as wind, hydro, and tidal energy has developed rapidly (Mankge et al., 2021; Hargreaves et al., 2020). Therefore, it is very important to develop large-scale and high-efficiency energy storage systems (Ani 2021; Züttel et al., 2022). As a large-scale power storage system, flow batteries have the characteristics of high capacity and wide application fields (environments), and will usher in a period of rapid development (Yang et al., 2021; Sankaralingam et al., 2021; Huang et al., 2021). In most flow batteries, iron-chromium flow batteries use low-cost Cr^3+^/Cr^2+^ pairs to reduce Cr^2+^ and Fe^3+^/Fe^2+^ pairs to oxidize Fe^3+^, respectively. Electrochemical redox reaction is carried out in Cr^3+^ electrolyte and acidic Fe^2+^ electrolyte (Zhang et al., 2020; Wu et al., 2021; Ahn et al., 2021). A typical iron-chromium flow battery system is shown in Figure 1, which consists of a point stack unit, an electrolyte, electrolyte storage and supply unit, and a management and control unit (Chen et al., 2020).

The key materials of flow batteries include electrodes, membranes, electrolytes, etc. Among them, graphite felt is the most commonly used electrode material in flow batteries (Zhang et al., 2017; Yue et al., 2010). At present, the activation methods of graphite felt are generally divided into two methods: adding oxygen-containing functional groups on the surface and introducing surface catalytic substances (Zhang et al., 2019). In terms of increasing the oxygen-containing functional groups on the surface, methods such as acidified K_2_Cr_2_O_7_ solution (Hassan et al., 2019), KMnO_4_ solution (Hassan et al., 2020), and laser coating modification (Daugherty et al., 2020) and aerogel modification (Jiang et al., 2019) are generally used. The introduction of oxygen-containing functional groups can not only increase the carbon and oxygen sites of the graphite felt modified electrode, increase the electrode activity, but also accelerate the charge transfer speed and improve the dynamic performance of the electrode (Na et al., 2018). The specific surface area of the felt (Jiang et al., 2019). In terms of introducing surface catalytic substances, electrostatic spraying graphene oxide coating (Anantha et al., 2021), rare earth composite oxide (Wang et al., 2020). and noble metal particles such as Pt and Ag are generally used as the main decoration (Xia et al., 2020; Lou et al., 2021), which can increase the current density and improve the current efficiency.

Indium and indium oxide are promising oxides (Xinyuan et al., 2021), and current research is mainly focused on the fact that indium and indium oxide can inhibit the hydrogen evolution reaction of the anode and improve the Coulombic efficiency of the battery. Leung et al. deposited zinc on a carbon composite electrode in a methane sulfonic acid medium and added 2 × 10^−3^ moldm^−3^ indium oxide as a hydrogen suppressor, and the energy efficiency was increased from 62 to 73% (Leung et al., 2011). Wang et al. used In^3+^ as an additive to improve the stability and performance of ICFBS, and their studies showed that In^3+^ can not only effectively inhibit the hydrogen evolution reaction, but also promote the reaction kinetics to a certain extent (Wang et al., 2021). The research on In^3+^ modified graphite felt electrodes has not been reported in detail. Therefore, it is of great significance to study the electro catalytic activity of In^3+^ modified graphite felt and its performance in ICRFBS.

## Experiment

### Preparation of Graphite Felt Electrodes

Graphite felt (GF, 5 mm, Gansu Haoshi Carbon Fiber Co., Ltd.) was heat-treated at 400°C for 2 h as the base material. Take three appropriate amounts of In_2_O_3_ powder and add them to a beaker of 3 M dilute hydrochloric acid respectively to prepare a 0.1, 0.2, and 0.3 M InCl_3_ solution. The following chemical reactions mainly take place in this process:

$${\text{In}}_{2}{\text{O}}_{3}+6\text{HCl}=2{\text{InCl}}_{3}+3{\text{H}}_{2}\text{O}$$

Three groups of graphite felts of the same size were immersed in 0.1, 0.2, and 0.3 M InCl_3_ solutions for 8 h, respectively. Then it was dried in a drying oven at 80°C for 15 h. The dried graphite felt was thermally activated in a medium-temperature experimental furnace at 400°C for 2 h. That is, the active graphite felt electrode for iron-chromium flow battery whose surface is coated with InCl_3_ is prepared.

### Characterization of Graphite Felt Electrodes

Scanning electron microscope (SEM) was used to observe the microscopic morphology of graphite felt, and X-ray energy dispersive spectroscopy (EDS) was used to determine the types and contents of elements on the surface of the samples. The N_2_ adsorption and desorption isotherms and the specific surface area (BET) and pore size distribution of each sample were measured by a specific surface area and pore size tester, and the pore size distribution was compared and analyzed by the BJH method (Chen et al., 2020).

### Electrochemical Measurements

Electrochemical performance was measured at room temperature using Wuhan Koster electrochemical workstation, and the flow battery electrolyte solution consisted of 1.0 M CrCl_3_ + 1.0 M FeCl_2_ + 3.0 M HCl solution. A three-electrode system was used for electrochemical measurement, 0.4 cm^2^ graphite felt was used as the working electrode, 1.0 cm^2^ platinum sheet was used as the counter electrode, and the reference electrode was a calomel electrode. Cyclic voltammetry tests were performed at a scan rate of 5 mV/s and a voltage range of −0.8–0.8 V. Electrochemical impedance measurements were performed in the frequency range from 0.01Hz to 100 kHz with an AC voltage amplitude of 5 mV and polarization potentials of 0.4V and −0.5 V, respectively.

## Results and Discussion

### Characterization of Physical Properties

**FIGURE 2:**
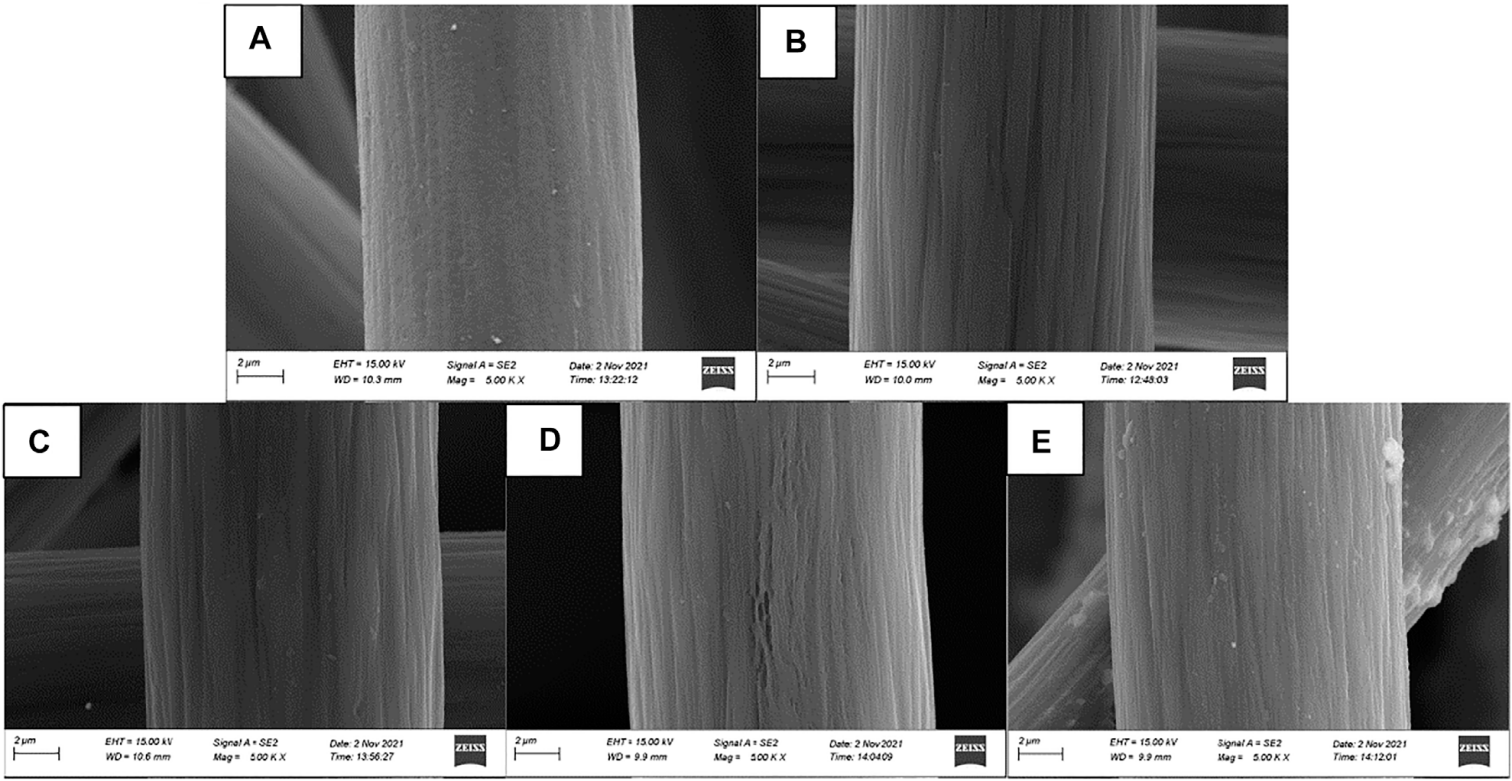
5.00 KX Scanning Electron Micrograph (SEM) **(A)**, untreated graphite felt **(B)**, heat treated graphite felt **(C)**, heat treated graphite felt impregnated with 0.1 M InCl_3_ solution **(D)**, heat treated graphite felt impregnated with 0.2 M InCl_3_ solution **(E),** heat treated graphite felt impregnated with 0.3 M InCl_3_ solution.

**FIGURE 3:**
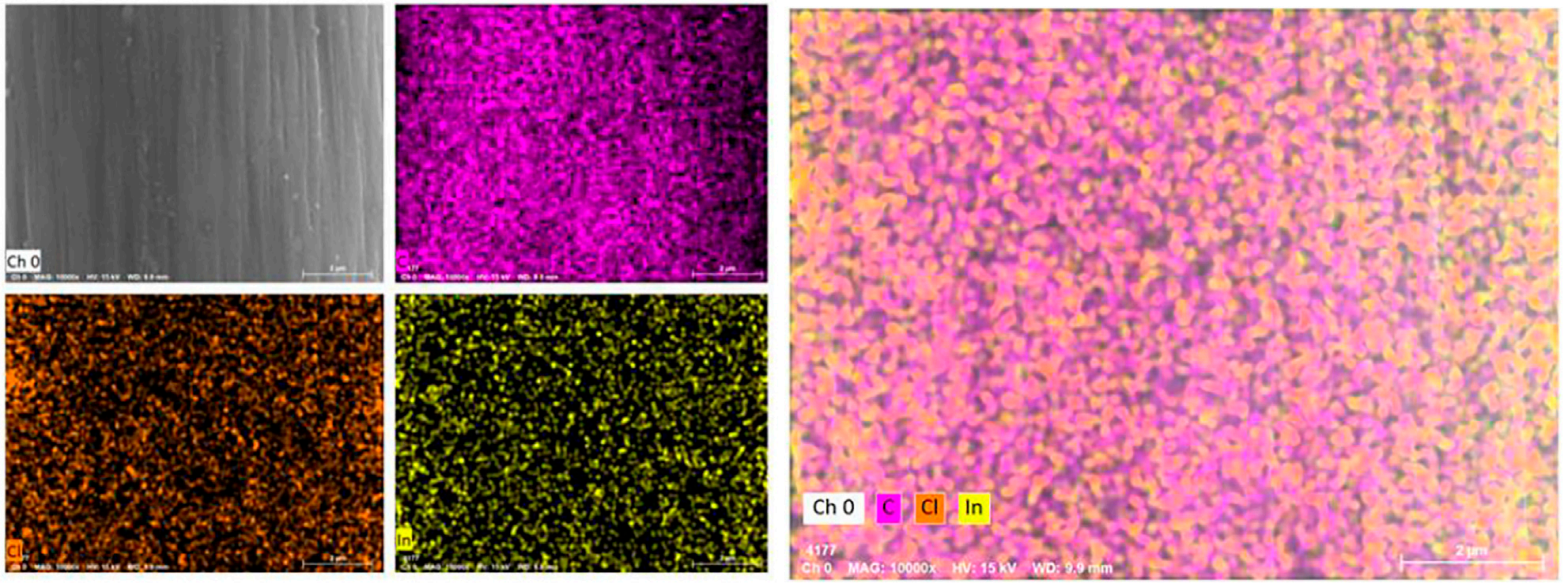
EDS analysis diagram of heat-treated graphite felt after immersion in InCl_3_ solution.

The surface morphology of graphite felt electrode observed by scanning electron microscope is shown in Figure 2. A small amount of impurities attached to the surface is the untreated graphite felt (Figure 2A), and a large number of deep “grooves” appeared along the fiber axis on the surface of the heat-treated graphite felt (Figure 2B). Figures 2C–E show the heat-treated graphite felt electrodes impregnated with InCl_3_ solutions of different concentrations, respectively. It can be seen that after immersion in the InCl_3_ solution, the depth of the “grooves” increases on the surface and is accompanied by the generation of irregular holes. When the concentration of InCl_3_ solution was 0.2 M (Figure 2D), the specific surface area of the graphite felt increased significantly to 3.889 m^2^/g, while the specific surface area of the untreated graphite felt was only 0.995 m^2^/g. In addition to the increase in the specific surface area of the graphite felt electrode, the EDS test results (Figure 3) showed that InCl_3_ was successfully coated on the fiber surface with uniform distribution, which may increase the activation point of the graphite felt electrode, which is beneficial to improve the performance of the electrode.

Take five pieces of graphite felt of the same size and put it into the electrolyte at the same time, observe its falling speed and position in three time periods of instant, 10 and 30 min, and judge its wettability.

**FIGURE 4:**
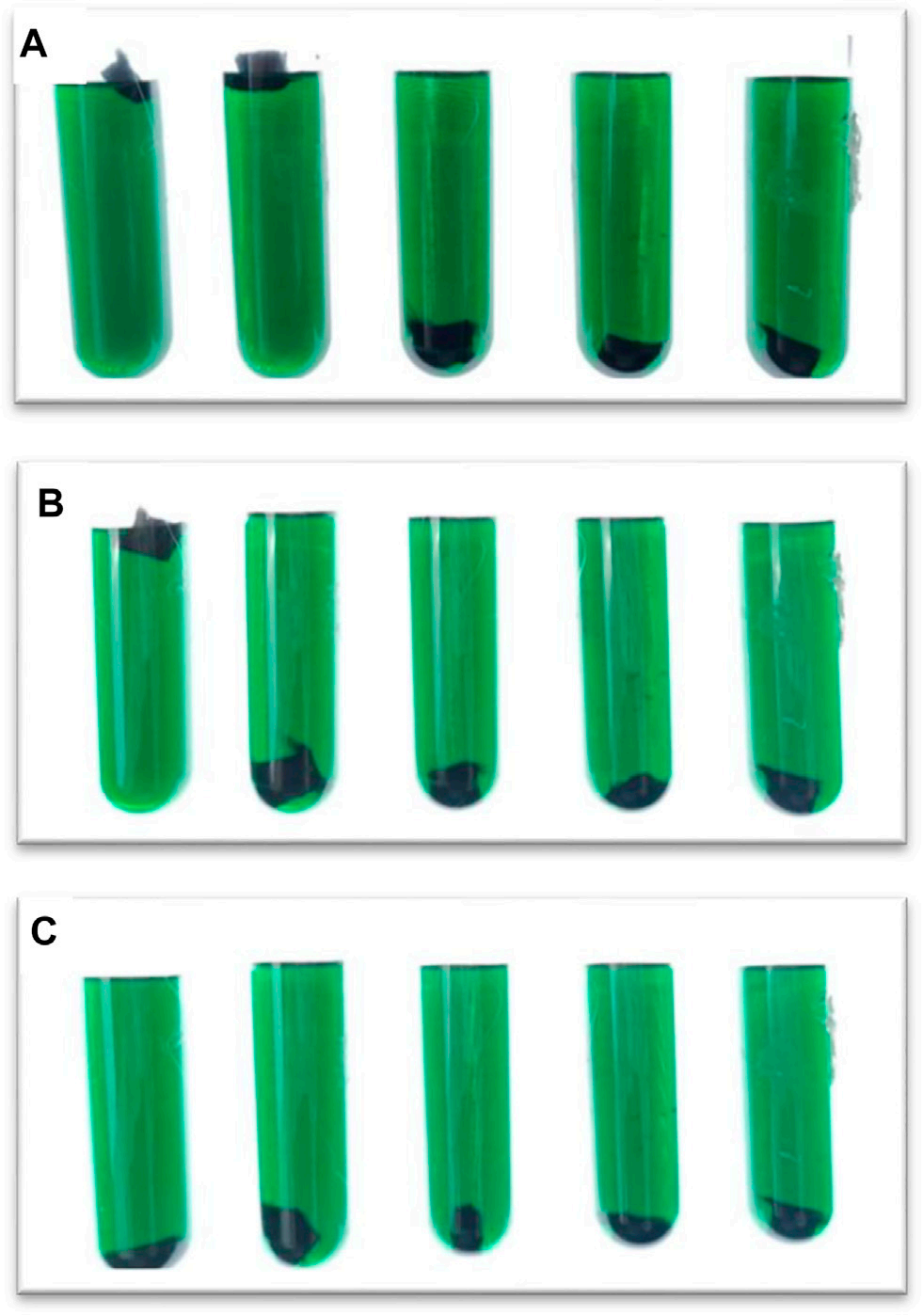
Samples from left to right are untreated graphite felt, heat-treated graphite felt, heat-treated graphite felt after immersion in 0.1 M InCl_3_ solution, heat-treated graphite felt after immersion in 0.2 M InCl_3_ solution, and heat-treated graphite felt after immersion in 0.3 M InCl_3_ solution, **(A),** momentary Contact; **(B),** soak for 10 min; **(C),** soak for 30 min.

It can be seen from Figure 4 that when the graphite felt is impregnated with InCl_3_ solution, its lipophilicity is significantly improved, and it will quickly sink into the electrolyte at the moment of contact, and the sinking speed of graphite felt impregnated with 0.2 M InCl_3_ solution will be slightly faster than Graphite felt impregnated with other concentrations of InCl_3_ solution. This method can measure the hydrophilicity of graphite felt, but the error is large. It can also be proved that the hydrophilicity of graphite felt is enhanced after being impregnated with InCl_3_ solution.

**FIGURE 5:**
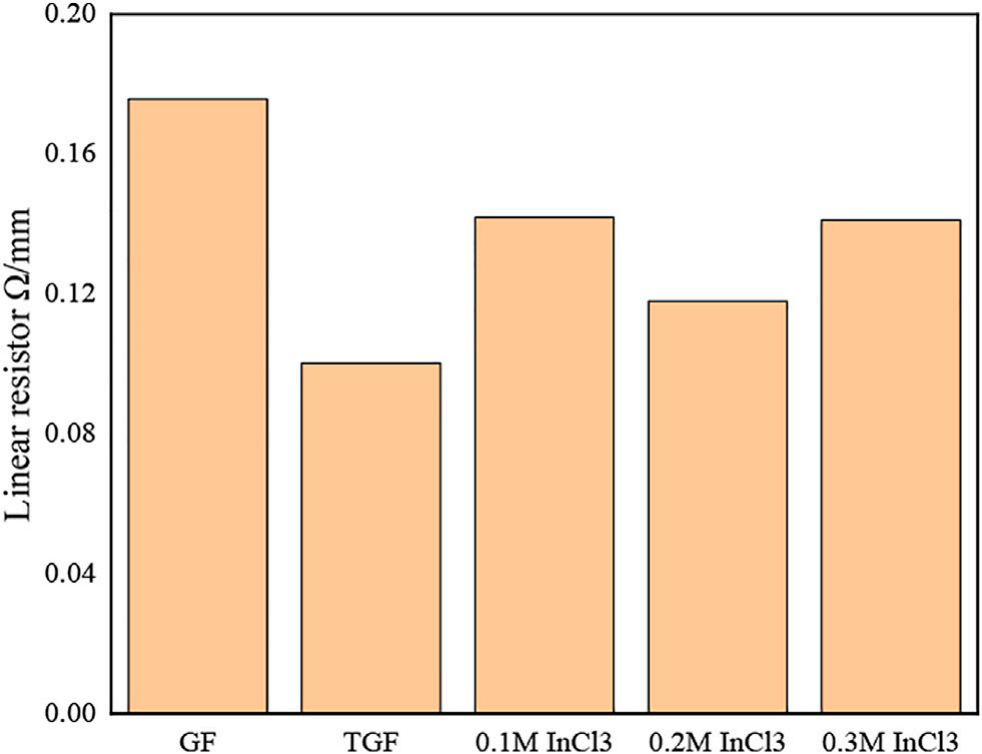
respectively GF; TGF; 0.1 M InCl_3_ solution impregnated heat-treated graphite felt; 0.2 M InCl_3_ solution impregnated heat-treated graphite felt; 0.3 M InCl_3_ solution impregnated heat-treated graphite felt line resistance.

In order to better understand the properties of graphite felt after immersion in InCl_3_ solution, we measured the resistance of each sample. Figure 5 shows the change of the line resistance value of each sample.

It can be seen from Figure 5 that the line resistance of GF is the largest, while the line resistance of TGF is slightly lower than that of GF, from 0.17 Ω/cm to 0.1 Ω/cm, and the reduction range is 41.17%. All have decreased, the decrease range is 17.64, 35.29, 17.65%, and it can be seen that the graphite felt impregnated with 0.2 M InCl_3_ solution has the lowest resistance.

### Electrochemical Characterization

**FIGURE 6:**
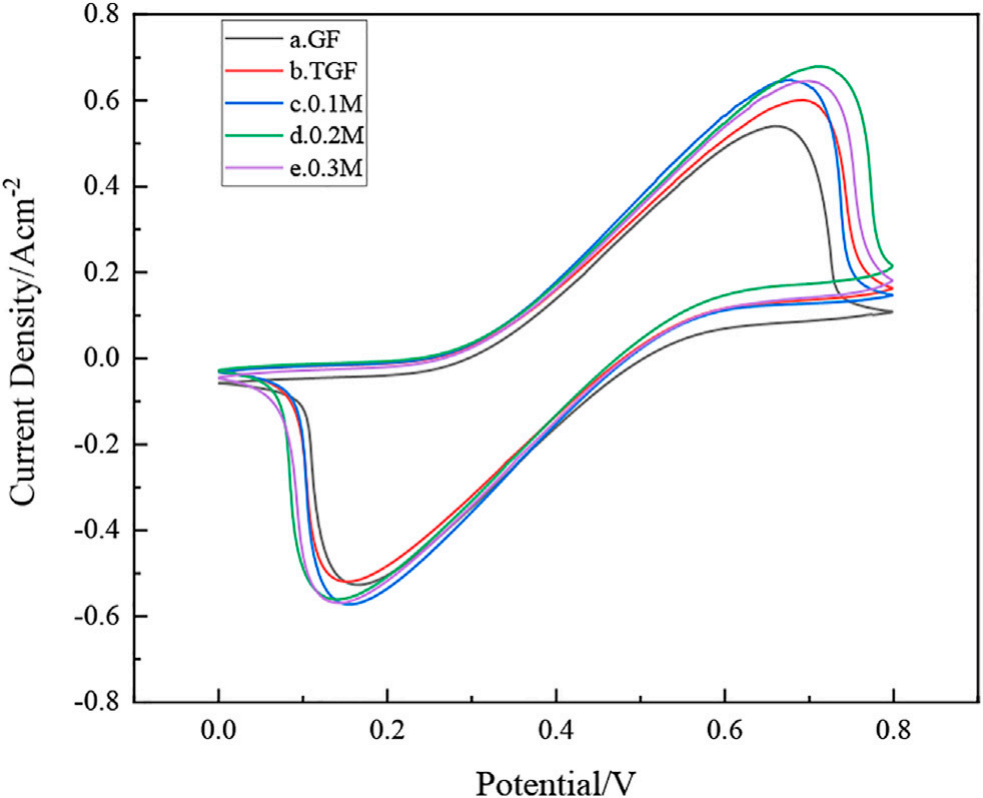
how’s samples **(a),** GF; **(b),** TGF; **(c),** heat-treated graphite felt after immersion in 0.1 M InCl_3_ solution; **(d),** heat-treated graphite felt after immersion in 0.2 M InCl_3_ solution; **(e),** heat-treated graphite felt after immersion in 0.3 M InCl_3_ solution, cycle Voltammetry curve.

As shown in Figure 6, the redox peak intensity of the cyclic voltammetry of the untreated graphite felt electrode is small, indicating that its electrochemical activity is low, and it is not suitable for direct use as an electrode material for iron-chromium batteries. The heat-treated graphite felt electrodes showed more obvious redox peaks after heat treatment and InCl_3_ solution immersion, indicating that the electrochemical performance of the heat-treated graphite felt electrodes was significantly improved compared with the untreated ones. Among them, the electrochemical performance of graphite felt electrode (d) after impregnation with InCl_3_ solution concentration of 0.2 M is the best, and the peak value of its wave peak is also stronger. It can be observed from Figure 6 that the peak current values of the positive electrode of the heat-treated graphite felt electrode and the graphite felt after being impregnated with InCl_3_ solution and then heat-treated are 587 mA, 642 mA, 692 mA, and 644 mA, all of which are greater than 524 mA. It can be proved that heat treatment after immersion in InCl_3_ solution can improve the electrochemical activity of the redox pair in Fe-Cr batteries.

**FIGURE 7:**
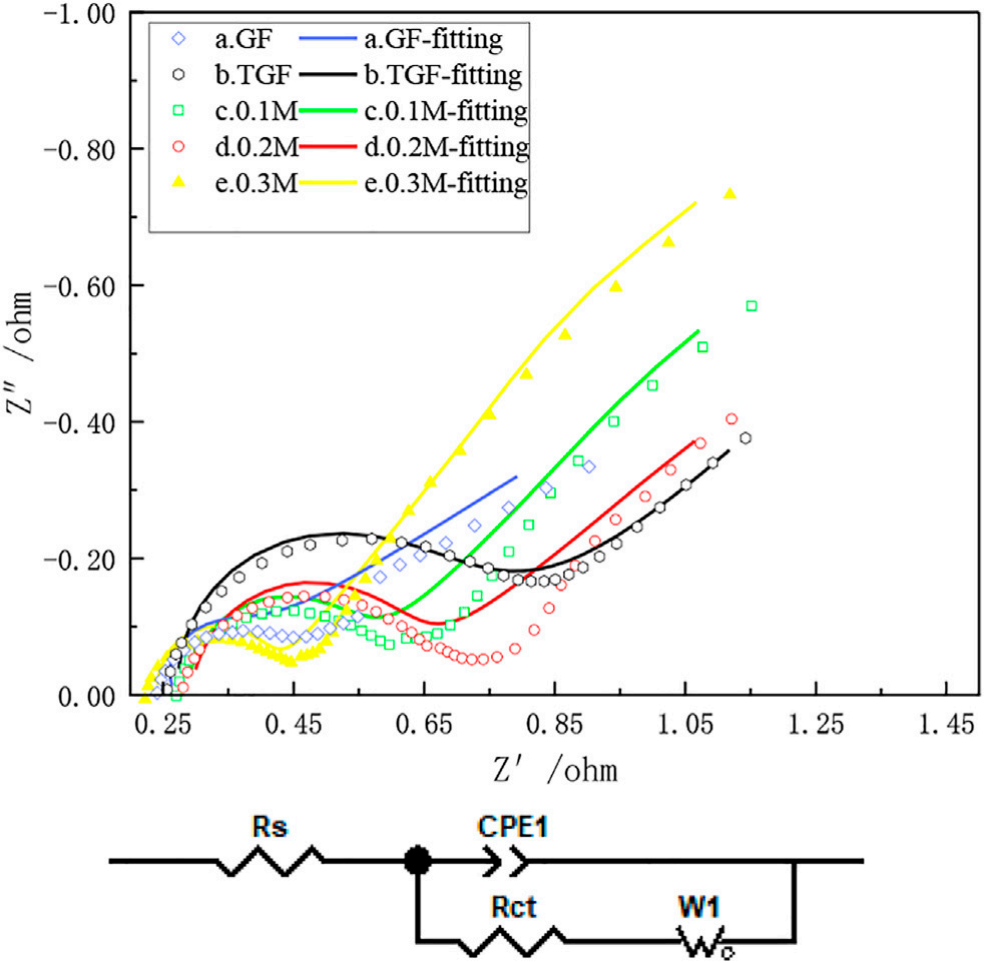
Samples **(a),** GF; **(b),** TGF; **(c),** heat-treated graphite felt after immersion in 0.1 MM InCl_3_ solution; **(d),** heat-treated graphite felt after immersion in 0.2 MM InCl_3_ solution; **(e),** heat-treated graphite felt after immersion in 0.3 MM InCl_3_ solution, electrochemical impedance Diagram and Equivalent Circuit Diagram.

Electrochemical impedance spectroscopy further analyzed the effect of heat treatment after immersion in InCl_3_ solution on the electrochemical performance of iron-chromium batteries. Figure 7 shows the Nyquist plots of a-e graphite felt electrodes. It can be observed from the figure that in all Nyquist diagrams, the semicircular part exists in the high frequency region, and the linear part exists in the low frequency region, which can indicate the interaction between iron ions and chromium ions on the graphite felt electrode. Redox reactions are affected by both the rate of charge transfer and the rate of diffusion. In Figure 7, when the electron transfer step at the electrode/electrolyte interface is the control step, the electrode process is corresponding to the semicircle arc located in the high frequency region, and the difficulty of the electron transfer is determined by the semicircle arc. Reflected by the radius of the arc (Rct), the smaller the resistance, the less difficult the transfer of electrons will be, and the smaller the radius will be; the diffusion coefficient of the reaction particles in the solution is the control step. Corresponding to the slash (Rs). Observing Figure 7, it can be seen that heat treatment after immersion in InCl_3_ solution can significantly reduce the charge transfer resistance of the iron-chromium redox couple, and the high-frequency arc radius is significantly smaller than that before treatment, indicating that heat treatment after immersion in InCl_3_ solution can accelerate the redox couple of iron-chromium. Reaction and charge transfer rates. However, it is easier for electrons to transfer on the graphite felt after being immersed in InCl_3_ solution and then heat-treated, that is, and the charge transfer resistance of the graphite felt is greatly reduced at this time, which further indicates that the heat-treated graphite felt electrode after being immersed in InCl_3_ solution is in iron. The electrochemical performance of chromium flow batteries has been greatly improved.

## Conclusion

In this paper, the graphite felt was immersed in InCl_3_ solution and then heat treated, so that indium ions were successfully introduced into the surface of the graphite felt to prepare an active electrode. The comprehensive SEM, EDS, BET, resistance, lipophilicity, and electrochemical test results can get conclusion:

1) In^3+^ was successfully coated on the surface of the fiber, and the distribution was uniform, which increased the activation point of the graphite felt electrode, which was beneficial to improve the performance of the electrode. When the In^3+^ concentration was 0.2 M, the specific surface area of the graphite felt increased significantly to 3.889 m^2^/g, while the specific surface area of the untreated graphite felt is only 0.995 m^2^/g.
2) The hydrophilicity of graphite felt impregnated with InCl_3_ solution is obviously enhanced, and it can be seen that the graphite felt impregnated with 0.2 M InCl_3_ solution has the lowest resistance.
3) Heat treatment after immersion in InCl_3_ solution can accelerate the redox reaction and charge transfer rate of iron-chromium charge, and the charge transfer resistance of the graphite felt after immersion in InCl_3_ solution is greatly reduced, thereby further improving its electrochemical performance.

## Data Availability

The original contributions presented in the study are included in the article/Supplementary Material, further inquiries can be directed to the corresponding author.
